# The role of the brain-derived neurotrophic factor genotype and parenting in early life in predicting externalizing and internalizing symptoms in children with attention-deficit hyperactivity disorder

**DOI:** 10.1186/1744-9081-10-43

**Published:** 2014-11-25

**Authors:** Subin Park, Bung-Nyun Kim, Jae-Won Kim, Yeon-Kyung Jung, Jin Lee, Min-Sup Shin, Hee Jeong Yoo, Soo-Churl Cho

**Affiliations:** Department of Psychiatry, Seoul National Hospital, Seoul, Republic of Korea; Deparment of Psychiatry and Behavioral Science, Seoul National University College of Medicine, Seoul, Republic of Korea; Department of Child and Adolescent Psychiatry, Seoul National University Hospital, Seoul, Republic of Korea

**Keywords:** BDNF, Parenting, ADHD

## Abstract

**Background:**

We aimed to determine whether early parenting is associated with externalizing and internalizing symptoms in children with attention-deficit hyperactivity disorder (ADHD) and whether such an association is affected by the brain-derived neurotrophic factor (BDNF) val66met polymorphism.

**Methods:**

The participants included 92 patients with ADHD aged 6–15 years. Measures of parenting in early life and externalizing and internalizing symptoms and the genotype of the BDNF Val66Met polymorphism were obtained.

**Results:**

The degree to which the baby’s autonomy was allowed was significantly and negatively correlated with the CDI scores in ADHD children (r = −0.38, p = 0.005). After adjusting for the child’s gender, the child’s age, the family’s gross annual income, and the maternal education level, there was a significant interaction for the BDNF genotype and mother’s positive feelings about caring in relation to the development of childhood anxiety/depression in ADHD children (F = 2.51, *p* = 0.011).

**Conclusions:**

Our results provide evidence of an interaction between the BDNF met allele and early parenting on the development of depression/anxiety symptoms.

## Background

Attention-deficit hyperactivity disorder (ADHD) affects 8% to 12% of school-aged children [1]. ADHD is characterized by symptoms of inattention and/or hyperactivity/impulsivity. With an estimated heritability of approximately 75%, ADHD is generally regarded as having a genetic basis [2]. However, the remaining phenotypic variance (25%) in ADHD has been largely attributed to environmental factors [3]. From the epigenetic perspective, environmental factors can also modulate gene expression and protein function in the brain [4]. Therefore, comprehensive investigations of environmental factors and gene-environmental interactions (G X E) are essential for a broad understanding of ADHD pathophysiology.

Given the wide heterogeneity and complex manifestations of the disorder, recent theoretical work has suggested the importance of a developmental perspective that views ADHD as a multi-factorial disorder with multiple, causal processes and pathways through development [5, 6]. Given the strong evidence in support of a biological basis of ADHD symptoms, there has been relatively little research exploring contextual variables within the family that may contribute to different outcomes for children with ADHD.

Indeed, much of the research literature has focused on the association between family factors and ADHD symptomatology [7–9], rather than on examining the broad spectrum of difficulties that children with ADHD often experience in development; these difficulties include externalizing problems (i.e., oppositional defiant disorder and conduct disorder) and internalizing problems (i.e., depression and anxiety).

A recent review focused on family characteristics associated with ADHD [10] provided a thorough examination of parenting variables in relation to the development of comorbidities and functional impairments in children with ADHD. The conclusions drawn from Deault’s review were that ADHD is associated with problematic family functioning, including higher rates of parental psychopathology and conflicted parent–child relationships; these relationship issues were exacerbated in children with comorbid oppositional and conduct problems. However, that review also revealed the paucity of studies that consider the role parents play in contributing to their children’s development in areas such as social and academic development, as well as the development of internalizing difficulties.

Although there is a paucity of studies that examined family factors contributing to the development of comorbid symptoms of ADHD (i.e., oppositional defiant disorder, conduct disorder, depression, and anxiety), many studies conducted in general population found that dysfunctional parenting was shown to their offspring’s externalizing and internalizing problems. For example, high level of behavioral control by parent is related to low levels of externalizing problems [11, 12] and low behavioral control and permissive parenting, and negative intrusive parenting behaviors linking to externalizing symptoms during early childhood [13] and also related to various kinds of maladjustment and conduct disorders [14, 15]. High level of psychological control(e.g., love withdrawal, guilt induction) and authoritarian parenting are associated with internalizing problems [15]. A high level of parental affection would decrease internal and external child problem behaviors [16], also sensitive parenting was associated with lower levels of child internalizing symptoms. [13] Psychological autonomy granting and acceptance by parent are associated with less depression and anxiety [17–19].

Brain-derived neurotrophic factor (BDNF) is a member of the neurotrophin superfamily, which includes growth factors that promote cell survival, differentiation and death. BDNF has key effects on the serotonergic [20], glutamatergic [21] and dopaminergic [22] neurotransmitter systems. BDNF is also involved in hippocampal long-term potentiation, which is related to learning and memory efficiency [23]. Given the large amount of BDNF protein in the central nervous system and its role in neurotransmission, a number of researchers have postulated the existence of a role for the BDNF gene (BDNF) in the pathogenesis and treatment response of different neuropsychiatric conditions, including depression, anxiety, and ADHD [24–26].

The BDNF gene, located on human chromosome 11p13, has a guanine-to-adenine single-nucleotide polymorphism (SNP) at nucleotide 196 (rs6265) that results in an amino acid substitution of methionine (Met) for valine (Val) at codon 66. This substitution changes the 5-prime proregion of the human BDNF protein and appears to lower depolarization-induced secretion of BDNF in the brain, leading to a decrease in available BDNF and a possible impairment of the central nervous system [27, 28]. The Met allele of the BDNF gene is associated with depression in the context of childhood adversity and genetic risk [29, 30].

In this study, we investigated the effect of early parenting on the development of ADHD and the relationship between parenting in early life and externalizing (i.e., oppositional defiant disorder and conduct disorder) or internalizing (i.e., social problems and depression/anxiety) symptoms during childhood among children with ADHD. Based on previous studies in general population [14, 15, 18, 19], we hypothesized that psychological autonomy granting and acceptance by parent might be associated with less depressive and anxiety symptoms and low behavioral control and permissive parenting might be associated with more oppositional defiant disorder and conduct disorder symptoms in children with ADHD. The interaction of parenting variables with the BDNF Val66Met polymorphism was also examined in relation to externalizing and internalizing symptoms.

## Methods

### Participants

Children and adolescents with ADHD were recruited from the Department of Psychiatry at the Seoul National University Hospital in Korea. The recruited children were between 6 and 15 years old and had been diagnosed with ADHD according to the DSM-IV criteria as ascertained by a child psychiatrist and a semi-structured interview. To diagnose ADHD and comorbid disorders, we used the Korean Kiddie-Schedule for Affective Disorders and Schizophrenia—Present and Lifetime Version (K-SADS-PL). The Korean version of K-SADS-PL was standardized by Kim *et al*. [31]. The Korean version of K-SADS-PL was reported to be valid and reliable for diagnosing major child psychiatric disorders in Korean children (consensual validity, kappa value = 0.695; test-retest reliability, kappa value = 0.755; sensitivity = 0.774; specificity = 0.947 for ADHD). The exclusion criteria for ADHD patients included the following: 1) a history of pervasive developmental disorder, mental retardation, bipolar disorder, psychotic disorder, obsessive compulsive disorder, or Tourette’s syndrome; 2) a history of organic brain disease, seizure disorder, or other neurological disorder; 3) an IQ below 70; 4) the presence of learning disabilities or language disorders; 5) the presence of major depressive disorder, anxiety disorder, or tic disorder requiring drug therapy; and 6) any previous course of stimulants or atomoxetine treatment lasting more than 1 year or occurring within the last 4 weeks. Exclusion of children who were on stimulants or atomoxetine is because these medications could alleviate externalizing and internalizing symptoms of ADHD, which might confound the results. To investigate the effect of early parenting on the development of ADHD, we enrolled healthy children and adolescents without ADHD between 6 and 15 years old as a control group. The exclusion criteria for the control group were the same as above, except for additionally excluding those with a past or an ongoing history of ADHD. For the control group, the K-SADS-PL and questionnaires on parenting variables were implemented, but comorbid symptom measurements and genotyping of the BDNF Val66Met polymorphism were not. The study protocol was approved by the institutional review board for human subjects at the Seoul National University Hospital. Detailed information about the study was given to parents and children, and written informed consent was obtained prior to study entry.

### Parenting variables

We evaluated mother-child interactions and maternal parenting style during infancy or toddlerhood using the self-report questionnaires developed by the Mater University Study of Pregnancy and its Outcomes (MUSP) group [32]. The MUSP is a prospective study of women, and their offspring, who received antenatal care at a major public hospital (Mater Misericordiae Hospital) in South Brisbane between 1981 and 1984. The mothers and children have been followed up prospectively with maternal questionnaires, covering a wide range of psychosocial and health characteristics of themselves, their partners and their children. When the children were 6 months, 5 years, and 14 years of age, the mothers were asked about the degree to which they agreed with statements about their subjective feelings towards care of the infant. The MUSP group found several findings regarding the effect of maternal attitudes or parenting practices on child’s psychopathological outcomes. For example, low maternal control at child age 5 has been reported to predict problematic patterns of adolescent alcohol consumption at age 14 [33], and maternal negative attitude towards the infant at 6 months has been shown to be an independent predictor of child behavioral outcomes at 5 years, especially the case for externalizing behavior [34]. The four domains of parenting attitudes or practices included in this study are listed below. The four domains included in this study are listed below.

#### Positive feeling about caring for the baby

Mothers were asked a series of 6 items about their feelings toward caring for their babies during the first year after birth (“Caring for baby is satisfying”; “Feel so angry sometimes I could smack my baby”; “My baby makes me too tired”; “My baby is so good”; “Sometimes I feel like hitting my baby”; “I feel fed up looking after my baby”). These items were assessed using a 5-point Likert-type scale (1 = strongly disagree to 5 = strongly agree). Scores of some items were reversed, and high scores represented positive feelings about caring for the baby. The scale produced a good reliability coefficient (Cronbach α =0.75) [35].

#### Time spent teaching baby

Mothers were asked a series of 4 items about the time spent teaching their babies during the first year after birth (“Try to encourage baby to be interested in what going on”, “My baby likes me talking to him/her”, “Spend a lot of time teaching baby to recognize things”, and “Love to play with my baby”). These items were assessed using a 5-point Likert-type scale (1 = strongly disagree to 5 = strongly agree). The scores for all of the items were reversed, and high scores represented high involvement in teaching the baby. The scale showed acceptable reliability (Cronbach α =0.65) [35].

#### Maternal parenting style: the baby is dominant

Mothers were asked a series of 3 items about their parenting styles with regard to their babies’ dominance during the first year after birth (“Always pick up baby as soon as he/she starts to cry”, “Think my baby should get his/her own way”, and “Don't allow my baby to rule my life”). Items were assessed using a 5-point Likert-type scale (1 = strongly disagree to 5 = strongly agree). Some items were reversed, and high scores represented a highly dominant baby.

#### Maternal authoritarian parenting style: degree of autonomy allowed

Mothers were asked a series of 3 items about their parenting styles with regard to their babies’ autonomy during toddlerhood (“Encourage child to go outside and play with other children”, “Expect child to disagree with me if he/she thinks I'm wrong”, and “Encourage child to do his/her own thing"). Items were assessed using a 5-point Likert-type scale (1 = strongly disagree to 5 = strongly agree). All of the items were reversed, and high scores represented high autonomy/low control. The scale showed a Cronbach’s α value of 0.48 [34].

### ADHD and comorbid symptom measurements

Children with ADHD completed the Korean version of the Children’s Depression Inventory [36]. The mothers completed Korean version of the ADHD Rating Scale-IV (ADHD-RS) [37] to assess the severity of ADHD symptoms and the Disruptive Behavioral Disorder Rating Scale according to the DSM-IV (DBDS) [38] to evaluate oppositional defiant disorder (ODD) and conduct disorder (CD) symptoms and the CBCL [39] to evaluate internalizing problems that commonly present in children with ADHD. Among the subdomains included in the CBCL, social problems and depression/anxiety were measured for this study.

### Genotyping of the BDNF Val66Met polymorphism

Genomic DNA was extracted from the blood (stored frozen) using a G-DEX II Genomic DNA Extraction Kit (Intron, Korea). The detection of a single nucleotide polymorphism was based on the analysis of primer extension products generated from the genomic DNA that was previously amplified using a chip-based MALDITOF mass spectrometry platform (Sequenom, Inc., California, USA). All primers in the PCR and homogenous mass extension reactions were designed using Assay Designer 3.1 (Sequenom, Inc.) (F: 5’- ACG TTG GAT GCA TCA TTG GCT GAC ACT TTC; R: 5’-ACG TTG GAT GCT TCA TTG GGC CGA ACT TTC) [40].

### Data analysis

Pearson correlation analyses were conducted to elucidate the relationships between parenting variables in toddlerhood and the ADHD and comorbid symptom manifestation in children with ADHD. We then performed linear regression analyses in which all parenting variables were concurrently entered along with the child’s gender, age, the family’s gross annual income, and the maternal education level. Next, we conducted multivariate modeling to examine the relationships among the parenting variables, the BDNF Val66Met genotypes, and the ADHD and comorbid symptoms; the child’s gender, the child’s age, the family’s gross annual income, and the maternal education level were included as covariates. A genotype-by-parenting variables interaction term was also included in the model. In these models, the genotype variable dichotomized subjects into two groups - children with the BDNF Val/Val genotype and children with another genotype, because several previous studies found that there were some differences in availability of BDNF protein [41] and clinical characteristics [42] between ADHD patients with Met allele and without Met allele. SPSS (version 21.0; SPSS Inc., Chicago, IL) was used to perform all statistical analyses, and a *p*-value less than 0.013 (=0.05/4 parenting variables) was considered to be significant. We also noted any finding with a *p*-value less than 0.05.

## Results

A total of 92 children with ADHD (72 males, 20 females, mean age 9.26 ± 2.67 years) and 41 healthy controls (27 males and 14 females, mean age 9.68 ± 2.83 years) participated in the study. Table 1 shows group-specific demographic and clinical characteristics. The age and gender distributions were not significantly different between ADHD and control subjects. Compared to control subjects, ADHD subjects showed lower scores on the positive feeling about caring for her baby (p = 0.013) and the degree of autonomy allowed (p = 0.034) and higher scores on the degree of the baby’s dominance (p = 0.039), but these differences were not statistically significant when adjusted with Bonferroni correction.

**Table 1:** **Characteristics of participants**

Variable	ADHD (N = 92)	Controls (N = 41)	***X*** ^2^ / t	p
Sex, male, n (%)	72 (78.3)	27 (65.9)	2.29	0.130
Age, mean (SD), years	9.26 (2.67)	9.68 (2.83)	−0.82	0.414
Maternal education, n (%)			0.97	0.324
College degree or more	56 (66.7)	23 (57.5)		
High school degree or less	28 (33.3)	17 (42.5)		
Income > $25,000, n (%)	65 (74.7)	25 (62.5)	1.94	0.164
Maternal age at pregnancy, mean (SD)	30.06 (4.26)	31.46 (3.45)	−1.81	0.072
Parenting variables, mean (SD)				
Positive feeling about caring for the baby	18.66 (4.49)	20.73 (4.09)	−2.53	0.013
Spending time teaching the baby	12.98 (2.73)	12.80 (3.57)	0.31	0.159
Parenting style: baby is dominant	10.47 (1.52)	9.80 (2.10)	2.08	0.039
Authoritarian parenting style: degree of autonomy allowed	9.76 (1.94)	10.55 (1.95)	−2.15	0.034
ARS, mean (SD)	24.80 (10.48)	4.87 (5.99)	13.40	<**0.001**
DBDS, mean (SD)				
ODD	4.91 (5.44)			
CD	1.29 (2.24)			
CBCL, mean (SD)				
Social problems	59.65 (9.64)			
Anxiety/depression	57.08 (8.90)			
CDI	10.77 (7.26)			

*Abbreviations:* *ADHD*, Attention-Deficit Hyperactivity Disorder; ARS, ADHD Rating Scale; *DBDS*, Disruptive Behavioral Disorder Rating Scale according to the DSM-IV; ODD, oppositional defiant disorder; *CD*, conduct disorder; *CBCL*, Child Behavior Checklist; *CDI*, Children’s Depression Inventory.

Bold: p <0.013.

Table 2 shows the correlations between parenting variables in toddlerhood and the ADHD and comorbid symptom manifestation in children with ADHD. The degree to which the baby’s autonomy was allowed was significantly and negatively correlated with the CDI scores (r = −0.38, p = 0.005). A mother’s positive feeling about caring for her baby and the degree to which the baby’s autonomy was allowed were negatively, but not significantly, correlated with the child’s anxiety/depression scores on the CBCL (r = −0.26, p = 0.019, each). The degree of the baby’s dominance was positively, but not significantly, correlated with the child’s ARS (r = 0.26, p = 0.018) and ODD (r = 0.26, p = 0.016) scores. These correlations remained even after adjusting for the child’s gender, the child’s age, the family’s gross annual income, the maternal education level, and other parenting variables (Table 3).

**Table 2:** **Correlations between parenting variables and externalizing or internalizing symptoms in children with ADHD**

	ARS scores		DBDS, ODD scores		DBDS , CD scores		CBCL, social problem scores		CBCL, depression / anxiety scores		CDI scores	
	r	p	r	p	r	p	r	p	r	p	r	p
Positive feeling about caring for the baby	−0.01	0.928	−0.03	0.780	−0.13	0.231	−0.18	0.107	−0.26	0.019	0.18	0.211
Spending time teaching the Baby	0.14	0.213	0.08	0.493	−0.04	0.696	−0.09	0.412	−0.11	0.318	−0.03	0.855
Parenting style: baby is dominant	0.26	0.018	0.26	0.016	0.10	0.355	0.11	0.316	<0.01	0.997	−0.05	0.743
Authoritarian parenting style: degree of autonomy allowed	−0.07	0.506	−0.09	0.424	<0.01	0.992	−0.16	0.164	−0.26	0.019	−0.38	**0.005**

*Abbreviations:* *ADHD*, Attention-Deficit Hyperactivity Disorder; ARS, ADHD Rating Scale; DBDS, Disruptive Behavioral Disorder Rating Scale according to the DSM-IV; *ODD*, oppositional defiant disorder; *CD*, conduct disorder; *CBCL*, Child Behavior Checklist; *CDI*, Children’s Depression Inventory.

Bold: p <0.013.

**Table 3:** **Associations between parenting variables and externalizing or internalizing symptoms in children with ADHD**

	ARS scores			DBDS, ODD scores			DBDS , CD scores			CBCL, social problem scores			CBCL, depression / anxiety scores			CDI scores		
	*B* (95% CI)	f^2^	p	*B* (95% CI)	f^2^	p	*B* (95% CI)	f^2^	p	*B* (95% CI)	f^2^	p	*B* (95% CI)	f^2^	p	*B* (95% CI)	f^2^	p
Positive feeling about caring for the baby	−0.21 (−0.78, 0.36)	0.05	0.490	−0.05 (−0.34, 0.24)	0.01	0.724	−0.09 (−0.22, 0.05)	0.51	0.190	−0.56 (−1.13, 0.01)	0.78	0.052	−0.60 (−1.09, −0.10)	0.71	0.019	0.46 (−0.09, 1.00)	0.19	0.096
Spend time teaching the baby	0.62 (−0.43, 1.66)	0.13	0.242	0.39 (−0.13, 0.92)	0.21	0.141	0.02 (−0.23, 0.26)	0.00	0.885	0.06 (−1.02, 1.14)	0.00	0.917	−0.05 (−1.00, 0.89)	0.00	0.910	0.27 (−0.82, 1.36)	0.02	0.614
Parenting style: baby is dominant	1.55 (−0.07, 3.17)	0.43	0.060	0.83 (0.01, 1.66)	0.45	0.049	0.18 (−0.21, 0.56)	0.20	0.361	0.89 (−0.69, 2.47)	0.16	0.264	0.00 (−1.39, 1.38)	0.00	0.998	0.09 (−1.33, 1.51)	0.00	0.898
Authoritarian parenting style: degree of autonomy allowed	−0.34 (−1.69, 1.01)	0.02	0.613	−0.31 (−1.05, 0.43)	0.06	0.405	0.09 (−0.20, 0.43)	0.06	0.591	−0.59 (−2.04, 0.85)	0.08	0.414	−1.20 (−2.47, 0.06)	0.31	0.062	−1.99 (−3.21, −0.77)	1.39	**0.002**

Associations (*B* coefficients) are with 1-unit increase in parenting variable scores.

In the linear regression analyses, all parenting variables were concurrently entered, along with child’s sex, age, maternal education level, and family’s gross annual income.

Abbreviations: *ADHD*, Attention-Deficit Hyperactivity Disorder; ARS, ADHD Rating Scale; DBDS, Disruptive Behavioral Disorder Rating Scale according to the DSM-IV; *ODD*, oppositional defiant disorder; *CD*, conduct disorder; *CBCL*, Child Behavior Checklist; *CDI*, Children’s Depression Inventory.

Bold: p <0.013.

Among the children with ADHD, the Val/Met genotype (50.6%) showed the highest frequency among the BDNF Val66Met polymorphism. The Val/Val genotype (27.1%) showed the next highest frequency and the remaining 22.3% of children had the Met/Met genotype. The genetic distributions of the Val66Met polymorphism of BDNF coincided with the expected values of the Hardy-Weinberg Equilibrium (*p* >0.05). There were no significant differences in comorbid symptom severity between ADHD children with the BDNF Val/Val genotype and those with the Val/Met or Met/Met genotypes (*p* >0.05).

After adjusting for the child’s gender, the child’s age, the family’s gross annual income, and the maternal education level, the interaction between the mother’s positive feelings about caring for her baby and the BDNF genotype was statistically significant only in the anxiety/depression variable model (F = 2.51, *p* = 0.011) (Table 4). The interactions of the other parenting variables with the BDNF genotype were not statistically significant in any of the comorbidity variable models (Data available on request).

**Table 4:** **ANCOVA models examining the main and interaction effects of a mother**’**s positive feeling about caring for her baby and the BDNF genotype on the depression**/**anxiety scores in children with ADHD**

	CBCL , depression / anxiety	
	F	p
Positive feeling about caring for the baby	2.51	**0.011**
BDNF genotype	1.73	0.198
Positive feeling about caring for the baby X BDNF genotype	2.54	0.024
Sex	5.94	0.020
Age	0.59	0.446
Maternal educational level	4.20	0.048
Family’s gross annual income	0.55	0.462

*Abbreviations:* ANCOVA, full analysis of covariance; BDNF, drain-derived neurotrophic factor; *ADHD*, attention-deficit hyperactivity disorder; *CBCL*, Child Behavior Checklist.

Bold: p <0.013.

Next, we conducted linear regression analyses using the anxiety/depression variable as the outcome and the mother’s positive feelings about caring for her baby as the predictor within each genotype; thus, separate models were created for subjects with the BDNF Val/Val and other genotypes. For the subjects with the BDNF Val/Met or Met/Met genotype, there was an association between the mother’s positive feelings about caring for her baby with the anxiety/depression scores on the CBCL (*B* = −0.59, 95% CI = −1.15 to −0.04, *p* = 0.035), although this association was not significant when adjusted with Bonferroni correction. However, for the subjects with the BDNF Val/Val genotype, no associations were found (*B* = −0.25, 95% CI = −1.55 to 1.06, *p* = 0.676) (Table 5).

**Table 5:** **Associations between a mother**’**s positive feeling about caring for her baby and the depression**/**anxiety scores by BDNF genotype in children with ADHD**

	BDNF Val / Val			BDNF Met / Val or Met / Met		
	***B*** ( 95 % CI )	f ^2^	p	***B*** ( 95 % CI )	f ^2^	p
Positive feeling about caring for the baby	−0.25(−1.55, 1.06)	0.02	0.676	−0.59(−1.15, −0.04)	0.80	0.035
Spend time teaching the baby	−1.17(−4.22, 1.88)	0.04	0.401	0.07(−0.89, 1.04)	0.00	0.877
Parenting style: baby is dominant	−1.17(−5.16, 2.81)	0.03	0.516	0.39(−0.97, 1.74)	0.04	0.567
Authoritarian parenting style: degree of autonomy allowed	−1.38(−4.52, 1.76)	0.08	0.340	−0.26(−1.73, 1.20)	0.01	0.718

Associations (*B* coefficients) are with 1-unit increase in parenting variable scores.

In the linear regression analyses, all parenting variables were concurrently entered, along with child’s sex, age, maternal education level, and family’s gross annual income.

*Abbreviations:* BDNF, drain-derived neurotrophic factor; ADHD, attention-deficit hyperactivity disorder.

## Discussion

The major findings of this study are that the degree to which the baby’s autonomy is allowed was associated with fewer depressive symptoms of ADHD children, and there was evidence of a G X E interaction for the BDNF Val66Met polymorphism and the mother’s positive feelings about caring for the baby in relation to the development of childhood anxiety/depression in ADHD children.

Children with ADHD experience significant difficulties with emotional regulation and are at greater risk for depression and anxiety [43]. However, relatively few studies have addressed the parenting factors associated with the depressive and anxious symptoms in children with ADHD [10]. Parental depression or anxiety symptoms, less warmth and more power assertiveness in parents, and an inconsistent parenting style were associated with children’s depressive symptoms, suggesting an interaction between parental psychopathology and parent–child relations [44–46]. With respect to children’s anxiety, maternal anxiety, over-protectiveness, and a lack of positive parenting were reported to be independent predictors of anxiety in children with ADHD [9]. Furthermore, anxious ADHD families have been described as more controlling, dependent, and discouraging of autonomy compared to non-anxious ADHD families [47]. These previous studies used a cross-sectional design to compare family characteristics or parenting variables between an ADHD group with depression or anxiety and an ADHD-only group. Such a cross-sectional design makes it impossible to identify a causal relationship between parenting variables and depression or anxiety problems. Although the notion that parents have an enduring influence on their children has intuitive appeal, the behavior and temperament of the child can also affect the parent–child interaction or the parenting style [48]. To overcome this limitation, a prospective longitudinal study is ideal. Although we did not use a prospective study and we gathered information on parenting and the children’s internalizing problems at a single time point, we could infer a casual relation between parenting variables and the children’s internalizing problems by asking questions focused on parenting in early life rather than current parenting practices. Consistent with previous cross-sectional studies [44, 46], we found that the encouragement of autonomy were associated with fewer depressive symptoms in children with ADHD.

Previous studies have found an interaction between the BDNF Met allele and early adversity on the development of depression symptoms in adulthood [27, 28]. However, it was not known whether such G X E effects exist in ADHD. We found that the BDNF Met allele moderated the association between less positive parenting in early life and the development of depressive symptoms in children with ADHD, suggesting that individuals with this allele may be more susceptible to early environmental influences. It is notable that the G X E interaction was significant only for the anxiety/depression scale of the CBCL and not for the CDI. Different raters (i.e. a child for the CDI and a parent for the CBCL) and different contents measured (i.e. the severity of depressive symptoms for the CDI and broader spectrum of depression and anxiety for the CBCL) may partially explain the different results between two scales.

Several studies have explored the role of family contextual factors in the development of oppositional or conduct problems in children with ADHD. These studies suggest that parental psychopathology and family conflict tend to be more strongly associated with oppositional and conduct symptoms than with inattentive or hyperactive symptoms. [10] Both parental psychopathology and parenting during early childhood (i.e., observed praise and positive affect) were predictors of the developmental course of oppositional or conduct problems [49, 50]. In the present study, we found one unique possible predictor of childhood oppositional behaviors, namely, the allowance of the baby’s dominance. It is notable that this factor was not associated with depression or anxiety but was only associated with the oppositional behaviors. In contrast, less positive feelings about caring for the baby and the discouragement of autonomy were not associated with externalizing problems but were associated with internalizing problems. This distinction between parenting factors that are associated with either internalizing or externalizing symptoms in ADHD suggests there are distinct developmental pathways for each comorbid disorder (i.e., depression, anxiety disorder, or ODD) in ADHD.

This study has several limitations. First, because the data on parenting variables in early life were based on the recollection of the mothers of the children, there is a potential for recall bias. In addition, the measures of parenting variables were drawn from the mother’s report only, without questioning the father or other informants. Second, it is not clear whether the child’s temperament influences the negative parenting attitude or whether the negative parenting contributes to the development of externalizing or internalizing problems. Third, data on the family history of psychiatric disorders and the psychopathology of mothers were lacking. Thus, the potentially confounding effects of genetic influences (i.e., heritability of depression) or maternal psychopathology (i.e., the effects of over-protectiveness in an anxious mother on the child’s anxiety) could not be examined. Fourth, there was no control group. To determine whether the influence of the BDNF genotype-parenting variable interaction on the child’s comorbid symptoms is specific to ADHD patients, we should have obtained parenting, symptomatic, and genetic data from healthy controls. Finally, the sample size of the present study was very small for genotypic analysis; thus, the results should be carefully interpreted and further work is needed to confirm the findings of this study.

Despite these limitations, our results provide evidence of an interaction between the BDNF met allele and early parenting on the development of depression/anxiety symptoms. However, owing to our small sample size, these results should be considered preliminary in nature and require careful interpretation. Additional prospective, controlled studies in a larger sample are warranted to evaluate the association between parenting in early life and the development of comorbid symptoms in ADHD as well as the effects of a G X E interaction.
